# Supplementary material for: Longitudinal analysis of Plasmodium sporozoite motility in the dermis reveals component of blood vessel recognition
Source: eLife. 2015 Aug 13;4:e07789. doi: 10.7554/eLife.07789 (PMC4594146; doi:10.7554/eLife.07789)
Supplement: Supplementary file 1. — Primer sequences used to generate and confirm the transgenic and mutant parasites described in this study. DOI: http://dx.doi.org/10.7554/eLife.07789.023 [file elife07789s001.docx]

**Table S1: Primers used for genotype analysis**

| **Diagnostic PCR to verify the selection marker free line 2204cl1** | |
| --- | --- |
| **Primer Name** | **Primer Sequence** |
| 4698 h*dhfr*, F | GTTCGCTAAACTGCATCGTC |
| 4699 y*fcu*, R | GTTTGAGGTAGCAAGTAGACG |
| 5515 3’int, mCherry, F | GCATGGACGAGCTGTACAAG |
| 5511 3’int, pb230p, R | AGTGACTTTCAGTGAAATCGC |
| 5510 5’int pb230p, F | GCAAAGTGAAGTTCAAATATGTG |
| 4958 5’int mCherry, R | GCATGAACTCCTTGATGATG |
| **Diagnostic PCR to verify the CSΔN parasite line** | |
| **Primer Name** | **Primer Sequence** |
| DP1 | AATGAGACTATCCCTAAGGG |
| DP2 | TAATTATATGTTATTTTATTTCCAC |
| 3'int-F | TGATTCATAAATAGTTGGACTTGATTT |
| 3'int-R | TCTTTTGGACATATATTCATTTTAGCA |
| CS-F | CCATTTTAGTTGTAGCGTCACTTTT |
| CS-R | TGTAAGCAATTCATTAGGATTTGT |
| **Diagnostic PCR to verify the TRAP-VAL parasite line**  *letters in brackets before the primer names refer to the letters shown in Fig. S3. | |
| **Primer Name** | **Primer Sequence** |
| (A) TX-1 TRAP 5’INT-FWD | CGATCAAATTAAAGAGCAAAAGTTCAC |
| (B) 5UTR hDHFRseqREV | CTTTGAGG GGTGAGCATTTAAAGC |
| (C) hDHFR-3UTRseq | GTCTCTTCAATGATTCATAAATAGTTGG |
| (E*) TX-2TRAP3’INT-REV | CAATCCAATAAGGATCCTAATAATTATTC |
| (D*) 5’UTRPbTRAP-REV | GCTAGCAGGATCCTATAAGGGAAAGGGAAAATGGGC |
| (F) SEQPbTRAP2-FWD | CAATTCGTATTATATGCGTATGTG |

*in the supplementary of Ejigiri et al. 2012 PloS Pathog primers D & E were inverted, an error that is corrected here.
